# Biochemical and physiological characterization of the GTP-binding protein Obg of *Mycobacterium tuberculosis*

**DOI:** 10.1186/1471-2180-11-43

**Published:** 2011-02-25

**Authors:** Smitha J Sasindran, Sankaralingam Saikolappan, Virginia L Scofield, Subramanian Dhandayuthapani

**Affiliations:** 1 Regional Academic Health Center and Department of Microbiology and Immunology, The University of Texas Health Science Center at San Antonio, Edinburg, Texas, 78541, USA

## Abstract

**Background:**

Obg is a highly conserved GTP-binding protein that has homologues in bacteria, archaea and eukaryotes. In bacteria, Obg proteins are essential for growth, and they participate in spore formation, stress adaptation, ribosome assembly and chromosomal partitioning. This study was undertaken to investigate the biochemical and physiological characteristics of Obg in *Mycobacterium tuberculosis*, which causes tuberculosis in humans.

**Results:**

We overexpressed *M. tuberculosis *Obg in *Escherichia coli *and then purified the protein. This protein binds to, hydrolyzes and is phosphorylated with GTP. An anti-Obg antiserum, raised against the purified Obg, detects a 55 kDa protein in immunoblots of *M. tuberculosis *extracts. Immunoblotting also discloses that cultured *M. tuberculosis *cells contain increased amounts of Obg in the late log phase and in the stationary phase. Obg is also associated with ribosomes in *M*. *tuberculosis*, and it is distributed to all three ribosomal fractions (30 S, 50 S and 70 S). Finally, yeast two-hybrid analysis reveals that Obg interacts with the stress protein UsfX, indicating that *M. tuberculosis *Obg, like other bacterial Obgs, is a stress related protein.

**Conclusions:**

Although its GTP-hydrolyzing and phosphorylating activities resemble those of other bacterial Obg homologues, *M. tuberculosis *Obg differs from them in these respects: (a) preferential association with the bacterial membrane; (b) association with all three ribosomal subunits, and (c) binding to the stress protein UsfX, rather than to RelA. Generation of mutant alleles of Obg of *M. tuberculosis*, and their characterization in vivo, may provide additional insights regarding its role in this important human pathogen.

## Background

GTP-binding proteins are found in all living organisms, and they play critical roles in fundamental processes such as cell proliferation, development, signal transduction and protein translation [1,2]. In general, these proteins are hydrolase enzymes that convert GTP into GDP, allowing transfer of the GTP terminal phosphate group to a target protein. As a consequence of this transfer, the highly conserved domains (G1, G2, G3, G4 and G5) of GTP-binding proteins undergo conformational changes that are detected by downstream effector proteins [3,4], leading to specific outcomes.

Comparison of bacterial genomes, across all taxa, has shown that at least eleven highly conserved GTP-binding proteins are present in prokaryotes [5]. Among these, the Obg/GTP1 subfamily of monomeric GTP binding proteins is of special significance, because these proteins exist not only in prokaryotes but also in eukaryotes [6]. The gene encoding Obg was first identified in *Bacillus subtilis *[7]. Obg orthologues were subsequently discovered in *Streptomyces griseus *[8], *Streptomyces coelicolor *[9], *Caulobacter crescentus *[10], *Echerichia coli *[11] and *Vibrio harveyi *[12]. While orthologues of Obg in *C. crescentus *and *V. harveyi *are known as CgtA, the orthologue of Obg in *E. coli *is called ObgE. Bacterial Obg display intrinsic GTPase activity and autophosphorylate with GTP, as does the eukaryotic signaling molecule Ras, which is a GTP-binding protein. Because of this, Obg has been considered to be a potential bacterial signaling molecule [8,13].

Several published studies have attributed diverse functions to Obg in different bacterial species. In *B. subtilis*, for example, Obg is necessary for the transition from vegetative growth to stage 0 or stage II of sporulation [14]. Sporulation is a complex process in this species and is controlled by multiple components including phosphorelay. It appears that Obg is one of the components that modulate the sporulation-related phosphorelay by an undefined mechanism [15]. In addition to its activity in *B. subtilis*, Obg plays critical roles in developmental events in other bacteria, e.g. aerial mycelium formation and sporulation in *Streptomyces griseus *[8] and *S. coelicolor *[9]. In these two species, sporulation has a tight relationship with changes in the intracellular GTP-to-GDP ratio, and bacterial Obgs are considered to be stress sensors for intracellular GTP-GDP changes reflecting energy balance in the cells. It has been proposed that high levels of Obg-GTP maintain vegetative division of sporulating bacteria and prevent sporulation, while high levels of Obg-GDP promote sporulation [9].

Obg is required for the activation of *B. subtilis *SigB in response to physical stress. This activation occurs via Obg's physical interaction with upstream Rsb regulators of SigB [16]. Further, the GTP-binding pocket of crystallized Obg of *B. subtilis *contains guanosine 5' diphosphate, 3' phosphate (ppGpp) [16]. ppGpp is a guanosine nucleotide known as an alarmone in bacteria. Alarmones are produced in response to amino acid starvation, and they act as signaling intermediates to slow cell growth or to initiate stress-induced differentiation pathways, including sporulation. In bacteria, the synthesis of ppGpp is performed by two enzymes, called RelA and SpoT [17-19]. In *E*. coli, SpoT is one of the proteins known to interact with Obg [20]. In *V. cholerae*, depletion of the Obg homologue CgtA results in a global gene expression pattern reflecting the low-nutrient stress reaction called the "stringent" response [21]. In *V. cholerae*, CgtA interacts with SpoT, and this interaction decreases SpoT activity leading to the repression of the stringent response [21]. Another interesting example of Obg's association with stress comes from the pathogen *Legionella pneumophila*, where its expression is elevated during intracellular survival [22].

Recent studies indicate that Obg associates with ribosomes of bacteria and interacts with ribosomal proteins. In *B. subtilis*, Obg coelutes with ribosomal proteins and interacts specifically with the ribosomal protein L13, a component of the 50 S ribosomal subunit [23]. The Obg orthologues of *C. crescentus *[24], *V. harveyi *[25] and *E. coli *[20,26] also cofractionate with the 50 S ribosomal subunit. Finally, bacterial Obg has also been implicated in chromosomal partitioning [11] and replication regulation [27].

*Mycobacterium tuberculosis *is an intracellular pathogen and causative agent of tuberculosis in humans. The recent emergence of multidrug (MDR-TB) and extremely drug resistant (XTR-TB) *M. tuberculosis *strains now poses serious threats to people in the developing world [27], and combating the disease requires the development of new anti-tuberculosis drugs. However, design and development of new drugs for TB largely depends upon the identification and characterization of novel drug targets in *M. tuberculosis*. The fact that Obg is an essential protein for growth in bacteria, including *M. tuberculosis *[28], and its association with ribosomes makes it a potential target for future antimicrobials [29,30]. Thus, this study was undertaken to understand the basic properties of Obg of *M. tuberculosis*.

## Results and Discussion

### Overexpressed *M. tuberculosis *Obg binds to, and hydrolyzes, GTP

**Figure 1:**
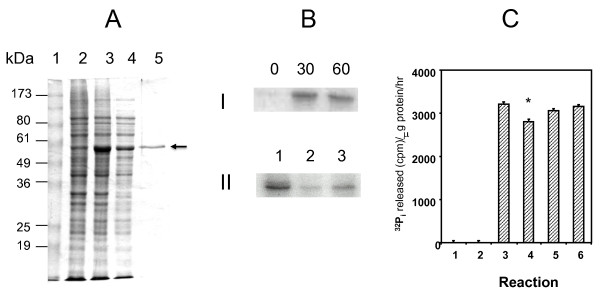
**Analysis of overexpressed Obg and its GTP binding and hydrolysis activities**. **A**. SDS-PAGE protein profile showing overexpression and purification of *M. tuberculosis *Obg. *E. coli *was grown in LB broth at 37°C, and lysates were prepared by sonication. Lane 1, Molecular markers; Lanes 2 and 3, extracts of *E. coli *strain BL21 carrying the overexpression plasmid pTBOBGE in the absence (Lane 2) and presence (Lane 3) of 1 mM IPTG; Lane 4, supernatant of *E. coli *lysate after 10,000 g centrifugation; Lane 5, His_10_-Obg after Ni-NTA affinity chromatography. The arrow points to the His_10_-Obg band. **B**. Autoradiogram of SDS-PAGE-separated *M. tuberculosis *His_10_-Obg after UV-crosslinking with [α^32^P]GTP. UV-cross-linking was performed by incubating 5 μg of His_10_-Obg with 10 μCi of [α^32^P]GTP in the binding buffer as described in the Methods section **I**. Crosslinking of His_10_-Obg with [α^32^P]GTP after 0, 30 and 60 minutes of exposure to UV light (256 nm). **II**. Crosslinking of His_10_-Obg with [α^32^P]GTP for 30 min without any additional GTP or ATP in the reaction mixture (Lane 1) or with 5 mM of unlabeled GTP (Lane 2), or with 500 mM of unlabeled ATP (Lane 3). **C**. GTPase activity of His_10_-Obg. GTP hydrolysis of His_10_-Obg was performed using [γ-^32^P] GTP at 37°C. The GTPase activity is expressed as ^32^P_i _released (cpm)/μg protein/hour. Columns indicate GTPase activity in the absence of [γ-^32^P]GTP and His_10_-Obg (Column 1), in the presence of His_10_-Obg alone (Column 2), in the presence of both [γ-^32^P]GTP and His_10_-Obg (Column 3), in the presence of [γ -^32^P]GTP, His_10_-Obg and 5 mM unlabeled GTP (Column 4), in the presence of [γ -^32^P]GTP, His_10_-Obg and 5 mM unlabeled GDP (Column 5) and in the presence of [γ-^32^P]GTP, His_10_-Obg and 5 mM unlabeled ATP (Column 6). * indicates value significant from column 3 (paired *t-*test P = 0.0163).

A single copy of the gene coding for Obg (*Rv2440c*) is present in the genome of *M*. *tuberculosis*, between the genes *proB *(*Rv2439c*) and *rpmA *(*Rv2441c*). The deduced amino acid sequence of the *M. tuberculosis *Obg protein shows significant similarities with the Obg proteins of *B. subtilis*, *S. coelicolor *and other bacterial species (Additional file 1). To study the properties of Obg of *M. tuberculosis*, the plasmid construct pTBOBGE was made to overexpress Obg in *E. coli*. Log phase *E. coli *cells (strain BL21) bearing the plasmid pTBOBGE were induced by IPTG to overexpress a protein that migrates at around 55 kDa in SDS-PAGE gels. This overexpressed protein, purified as detailed in the Methods section, showed a single protein in SDS-PAGE (Figure 1A). This was designated as His_10_-Obg, to distinguish it from the native, normally expressed Obg protein in *M. tuberculosis*.

To verify whether the overexpressed Obg of *M. tuberculosis *can interact with GTP, we performed GTP-UV-crosslinking experiments [31]. The autoradiogram in Figure 1B shows that His_10_-Obg binds physically to [α^32^P]-GTP. Exposure of the reaction mixtures to UV irradiation for 0, 30 and 60 min revealed that binding of GTP with His_10_-Obg is increased between 0 and 30 min of exposure, but not after 30 min (Figure 1B). When the reactions were performed in the presence of unlabeled GTP (5 mM), crosslinking of His_10_-Obg to GTP is inhibited, while addition of large amounts of unlabeled ATP (500 mM) have little effect on His_10_-Obg binding with labeled GTP (Figure 1B). This observation adds to existing evidence that *M. tuberculosis *Obg has an inherent specificity for guanine nucleotides, as do the Obg orthologues in *C. crescentus *[32], *B. subtilis *[13] and *S. griseus *[8].

To determine whether the overexpressed Obg can hydrolyze GTP, we incubated His_10 _-Obg with radiolabeled GTP ([γ-^32^P] GTP), and measured the release of phosphate (^32^P_i_) after 3 hours. Figure 1C shows that His_10_-Obg readily hydrolyzes GTP, and that this hydrolysis is inhibited by the addition of unlabeled GTP (5 mM), indicating that unlabeled GTP competes with labeled GTP for the enzyme. Addition of unlabeled ATP (5 mM) has no effect on the hydrolysis of labeled GTP (Figure 1C), indicating that Obg hydrolyzes specifically GTP. The effect of cold GTP in inhibiting the hydrolysis of radiolabeled GTP was not as pronounced as its effect in inhibition of GTP crosslinking (Compare Figure 1B and Figure 1C). This is most likely due to the differences in the positions of the radiolabeled phosphates used in these two reactions. While the reaction mixture in the crosslinking experiment (Figure 1B) had 10 μCi (0.033 μM) of [α-^32^P] GTP, the reaction mixture in the hydrolysis experiment had 25 μCi (0.040 μM) of [γ-^32^P] GTP. In addition, the incubation times for these two experiments were different (1 h for GTP crosslinking vs. 3 h for GTP hydrolysis).

### Autophosphorylation of His_10_-Obg

**Figure 2:**
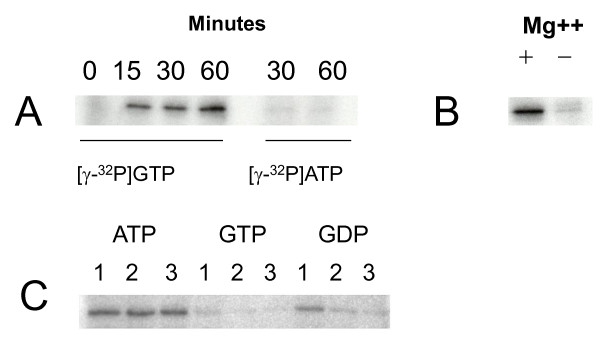
**Autoradiogram of SDS-PAGE-separated *M. tuberculosis *His_10_-Obg after autophosphorylation**. Autophosphorylation reactions were set up by incubating 5 μg of His_10_-Obg with 10 μCi of [γ-^32^P] GTP in autophosphorylation buffer, as detailed in the Methods section. **A**. Autophosphorylation of His_10_-Obg by [γ-^32^P] GTP or [γ-^32^P]ATP after 0, 15, 30 and 60 minutes of incubation at 37°C. **B**. Autophosphorylation of His_10_-Obg by [γ-^32^P]GTP in the presence (+ lane) and absence of (- lane) 1.5 mM MgCl_2 _. **C**. Autophosphorylation of His_10_-Obg by [γ-^32^P]GTP in the presence of 5 mM (Lane 1), 50 mM (Lane 2) and 500 mM (Lane 3) ATP; 5 mM (Lane 1), 50 mM (Lane 2) and 500 mM (Lane 3) of GTP; 5 mM (Lane 1), 50 mM (Lane 2) and 500 mM (Lane 3) of GDP.

Autophosphorylation by GTP is a defining characteristic of eukaryotic GTP-binding proteins, e.g. Ras [33], and of prokaryotic GTP-binding proteins, including Era of *E. coli *[34] and Obg of *B. subtilis *(22). We therefore asked whether His_10_-Obg of *M. tuberculosis *is autophosphorylated by GTP. Figure 2A shows that purified His_10_-Obg from *M. tuberculosis *is autophosphorylated by [γ-^32^P] GTP, in a time-dependent manner. This autophosphorylation is fully dependent upon Mg^2+ ^ions, since reactions conducted in the absence of MgCl_2 _in the buffer show almost zero phosphorylation activity (Figure 2B). By contrast, no autophosphorylation of His_10_-Obg occurs with [γ-^32^P] ATP, even after 60 min of incubation. Further, addition of unlabeled ATP to the reaction mixture fails to produce any effect on His_10_-Obg phosphorylation with [γ-^32^P] GTP (Figure 2C). As expected, both unlabeled GTP and GDP significantly affect the phosphorylation of [γ-^32^P] GTP from His_10_-Obg (Figure 2C), indicating that both molecules serve as competitors for the phosphorylation site. The eukaryotic Ras protein, which is encoded by the p21^ras ^oncogene, controls cell proliferation, cell stress signaling and apoptosis. The autophosphorylaiton of Ras is independent of its GTPase activity [33], which means that GTP hydrolysis and GTP phosphorylation of Ras occur at two different sites. At present it is unclear whether GTP hydrolysis and GTP-mediated autophosphorylation are independent events for prokaryotic Obgs, and no one has identified a phsophorylation site on any Obg molecule.

### Expression of *M. tuberculosis *Obg is growth-dependent, and Obg is associated with the membrane fraction

**Figure 3:**
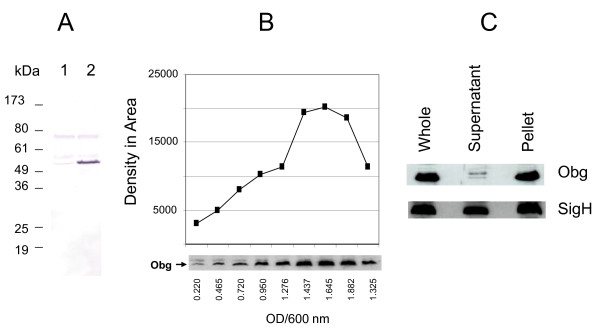
**Immunoblot analysis of Obg of *M. tuberculosis***. **A**. Immunoblot analysis of Obg from *M. tuberculosis *strains harboring plasmids. *M*. *tuberculosis *strains were grown in 7H9-OADC-TW broth at 37°C to early log phase and lysates prepared using a bead beater and separated (100 μg protein for each lane) on SDS-PAGE. The immunoblots were probed with anti-Obg antiserum (1:500 dilution) followed by alkaline phosphatase labeled anti-rabbit IgG (1:1000 dilution, Zymed). The antibody-incubated blots were then developed with NBT/BCIP substrates. Lane 1, *M. tuberculosis *carrying the plasmid pMV261(empty vector control); Lane 2, *M. tuberculosis *carrying the plasmid pMVOBG (plasmid overexpressing Obg). **B**. Immunoblot analysis of Obg at different growth points in *M*. *tuberculosis *culture. Wild type *M. tuberculosis *was grown in 7H9-OADC-TW broth at 37°C. Lysates were prepared from wild-type *M. tuberculosis *grown to different ODs at 600 nm, separated (200 μg protein for each lane) on SDS-PAGE, and probed with anti-Obg antiserum (1:500 dilution) followed by peroxidase-labeled anti-rabbit IgG (1:10,000 dilution, Sigma). The blots were developed with an ECL kit (Amersham) and autoradiographed. "Obg" indicates the Obg protein reacting with anti-Obg antiserum. Values below each band indicate the OD value at 600 nm at the time of harvest. The graph above the bands gives the levels of Obg, based on density of the bands using Image J software. **C**. Immunoblots of Obg in separated soluble vs membrane fractions of *M. tuberculosis *lysates. The bacteria were grown in 7H9-OADC-TW broth at 37°C to mid-log phase. Lysates were prepared using a bead beater, and the soluble and pellet fractions separated by centrifugation. The protein fractions (200 μg protein for each lane) were separated by SDS-PAGE, blotted and probed with anti-Obg antiserum (1:500 dilution) (marked as Obg) or anti-SigH antiserum (1:1000 dilution) (marked as SigH), followed by peroxidase-labeled anti-rabbit IgG (1:10,000 dilution, Sigma). The blots were developed with an ECL kit (Amersham) and autoradiographed. In the figure, lanes labeled Whole, Supernatant and Pellet represent extracts of whole *M. tuberculosis*, of the 49,000 g supernatant, and of the 49,000 g pellet, respectively.

In the sporulating bacterium *S. coelicolor*, the expression of Obg is regulated developmentally and is linked to the onset of sporulation [9]. By contrast, no such change in expression of Obg occurs in *C. crescentus*, although it also has a clear developmental cycle involving sporulation [10]. *M. tuberculosis *is a slow growing bacterium which exhibits neither sporulation nor a developmental cell cycle during its growth in culture. To determine whether the expression of Obg changes during the growth of *M. tuberculosis *in culture, we developed a rabbit anti-Obg antiserum against *M. tuberculosis *His_10_-Obg, and used it in Western blots of *M. tuberculosis *protein extracts. This antiserum detects multiple bands in immunoblotted extracts of *M*. *tuberculosis*, particularly at 55 kDa and 75 kDa. To confirm that the 55 kDa protein reacting with anti-Obg antiserum is in fact Obg, we cloned the coding region of Obg downstream of the *hsp60 *promoter in the plasmid pMV261, and transformed the resulting construct (pMVOBG) into *M*. *tuberculosis *to overproduce Obg. Figure 3A shows that protein extracts of *M. tuberculosis *strains harboring plasmid pMVOBG, but not strains bearing the vector plasmid pMV261, reveal strong 55 kDa protein bands, indicating that the protein at 55 kDa is Obg. Further analysis revealed that the 75 kDa band was a false reactivity due to the second antibody, and that it is not an Obg protein.

Notably, Obg expression does change in cultures of *M. tuberculosis *over the course of cell growth. Obg expression is markedly increased from early log phase to the stationary phase, with a drop in expression at late stationary phase (Figure 3B). Comparison of the Obg band densities discloses that expression of Obg at later growth phases (1.645 OD_600 nm _) is approximately five fold higher than it is at earlier phases (0.220 OD_600 nm_), even before the drop in expression at late stationary phase. Together these results indicate that the expression of Obg in *M. tuberculosis *is growth-regulated, being increased as the cells begin rapid division in the log phase, and maintained at high levels until late in the stationary phase. However, whether increased levels of Obg with increased growth of *M. tuberculosis *is due to increased expression of Obg, or to accumulation of Obg, remains to be determined. Obg expression in *E. coli *is also high in log phase growth, but decreased in the stationary phase [26].

In *S. griseus *[8] and *E. coli *[11], Obg and its orthologues are found in both the cytoplasmic and membrane fractions. In *B. subtilis*, however, Obg is mainly associated with the cytoplasm [23]. To determine where Obg resides in *M. tuberculosis*, we isolated soluble and membrane fractions from whole bacteria, and subjected them to immunoblot analysis. Figure 3C shows that Obg is associated mostly (over 90%) with the membrane fraction, although detectable amounts are also present in the soluble fraction. In contrast, SigH of *M. tuberculosis*, which was used as a control here, exhibits almost equal distribution between these two fractions. It has been reported that membrane fraction-bound Obg in *S. coeliocolor *[9] and in *E. coli *[11] is lost from this fraction if the extraction buffer contains 5 mM EDTA. The buffer we use for *M. tuberculosis *membrane preparations has 10 mM EDTA, however, and Obg is associated with this fraction whether or not EDTA is present (not shown). The EDTA-resistant association of *M. tuberculosis *Obg to the membrane fraction may reflect a function associated with signaling, and involving divalent cations. Interestingly, Obg is absent from detergent-extracted *M. tuberculosis *membrane [35] and cell wall [36] proteins, suggesting that Obg's association with the membrane may be due to its interaction with other membrane protein(s).

### *M. tuberculosis *Obg associates with ribosomal fractions

**Figure 4:**
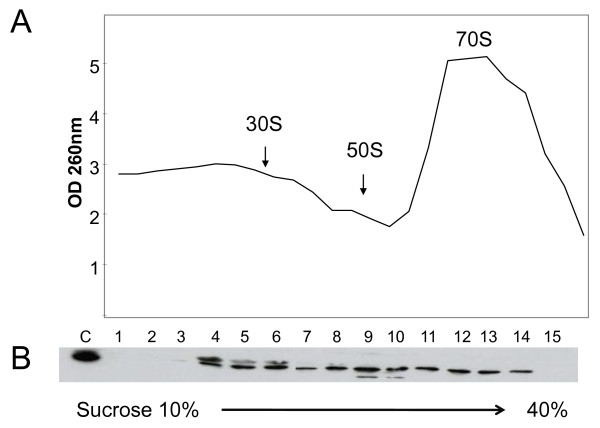
**Obg cofractionation with ribosomal subunits**. *M. tuberculosis *was grown in 7H9-OADC-TW broth at 37°C, and lysates prepared using a bead beater. About 500 g protein was separated in 10-40% sucrose gradient. **A**. The ODs of the separated fractions were measured (manually) at 260 nm. **B**. The proteins in the fractions were then precipitated with ethanol and separated on SDS-PAGE, transferred to nitrocellulose membranes, and probed with anti-Obg antiserum (1:500 dilution), followed by peroxidase-labeled anti-rabbit IgG (1:10,000 dilution, Sigma). The blots were developed with an ECL kit (Amersham) and autoradiographed. Lane C is a whole-cell extract from *M. tuberculosis*. Lanes 1-15 represent fractions from the top (10% sucrose) to the bottom (40% sucrose) of the sucrose gradient. Fraction 16 was not analyzed in immunoblot.

In *B. subtilis *[23], *C. crescentus *[24], *V. harveyi *[25] and *E. coli *[20,26], Obg has been shown to be associated with ribosomes. In these species, Obg orthologues cofractionate primarily with the 50 S ribosomal subunit [23,24,26]. To determine whether this is also true of *M. tuberculosis *Obg, we isolated ribosomes from *M. tuberculosis *using sucrose gradient centrifugation, as detailed in the Methods section (Figure 4A). Immunoblots of the separated ribosomal fractions (Figure 4B) show that Obg is present in all three (30 S, 50 S and 70 S) ribosomal fractions, in more or less equal amounts. By contrast, this discrepancy does not appear to be due to improper separation of ribosomal proteins in our sucrose gradient, because analysis of the ribosomal fractions in SDS-PAGE reveals that separation of proteins occurred in the expected line (Additional file 2). The Obg/CgtA of *E. coli *and *C. crescentus *has been shown to interact with specific 50 S ribosomal proteins, and it is the opinion of the investigators in this area that Obg plays a critical role in ribosome assembly. Evidence in support of this hypothesis has been provided with strains producing mutant Obg/CgtA. For example, *C. crescentus *[37] and *E. coli *[26] strains expressing mutated Obg have perturbed ribosomal protein profiles. A genetic basis for the involvement of Obg in ribosomal assembly has also been provided in *E. coli *by studies in which Obg was overexpressed in an *rrmJ *mutant strain [38]. Notably, *rrmJ *encodes an RNA methyltransferase which is involved in the assembly of 50 S ribosomes [38]. In line with these observations in bacteria, Obg homologues in yeast (Mtg2P) [39] and mice (Nog1) [40] also show association with ribosome maturation and assembly. Interestingly, in our studies shown here in Figure 4, lanes 4-6 (30 S region) and lanes 9 and 10 (50 S region) show an additional band above and below Obg, respectively. We do not know whether these bands represent modified forms of Obg. Work in progress includes studies toward identification of these bands.

### *M. tuberculosis *Obg interacts with UsfX

Scott et al [41] were the first to observe that *B. subtilis *Obg interacts with upstream regulators of the stress sigma factor SigB. In this respect, this bacterium's Obg resembles *B. subtilis *RsbT and RsbW, both of which also interact with SigB in this species [41]. More recently, the Obg proteins of *E. coli *[20] and *V. harveyi *[21] have been shown to interact with SpoT, a stringent response regulator. Since *SigB, RsbW *and *SpoT*-related genes are present in *M. tuberculosis*, we asked whether *M. tuberculosis *Obg interacts with any or all of these proteins, in the yeast two-hybrid system. The *M. tuberculosis *genes coding for Obg (*Rv2240c*), UsfX (homologue of RsbW, *Rv3287c*), SigF (homologue of SigB of *B. subtilis*, *Rv3286c*) and RelA (a stringent response regulator related to SpoT, *Rv2853c*) were cloned in yeast vectors, and transformed into the yeast strain AH109. Table 1 shows that *M. tuberculosis *Obg strongly interacts with UsfX, but not with the SpoT-related RelA protein. The strength of this interaction is comparable to the interaction of *M. tuberculosis *UsfX with its cognate sigma factor SigF. In the same experiment, we looked for interaction of *M. tuberculosis *Obg with various other putative anti-anti sigma factors that we have described earlier for this bacterium [42], including RsbU (*Rv1364c*), RsfA (*Rv1365c*), RsfB (*Rv3687c*), Rv0516c, Rv1904 and Rv2638. However, we observed no significant interaction of Obg with any of the above anti-anti sigma factors (data not shown), indicating that the interaction of *M. tuberculosis *Obg is limited to UsfX. In light of the known stress response role of UsfX [43], its specific interaction with Obg suggests that Obg plays a role in the *M. tuberculosis *stress response.

**Table 1:** Interaction of Obg with stress related proteins in the yeast two-hybrid system.

	*Plasmids	SD Minimal Medium			Mel-l (α-gal) in SD plates	Mel-1 (α-gal) in SD broth**
				
		-Leu/ -Trp	-His/ -Leu/-Trp	-Ade/-His/ -Leu/-Trp		
1.	pGADT7-T	+	+	+	+++	3.512 ± 0.709
	pGBKT7-53					
2.	pGADT7-T	+	-	-	-	-
	pGBKT7-Lam					
3.	pGA3287c	+	+	+	++	2.367 ± 0.354
	pGB3286c					
4.	pGA3287c	+	+	+	++	2.172 ± 0.448
	pGB2440c					
5.	pGA2853c	+	-	-	-	-
	pGB2440c					

**Abbreviations**. SD, synthetic drop out medium; Ade, Adenine; His, Histidine; Leu, Leucine; Trp, Tryptophan; Mel-1, α-galactosidase.

*1-5 indicate plasmids cotransformed into yeast strain AH109 (*HIS3, ADE2, MEL1*) (Clontech).

**α-galactosidase (Mel-1) expressed as Mean ± SD milli units/A600. Plasmids are described in Table 3.

In *B. subtilis*, the activation of SigB in response to stress depends upon its association with, and dissociation from, of RsbW. In turn, this is governed by the phosphorylation state of RsbW [44]. The UsfX protein of *M. tuberculosis *is believed to have similar interaction with its cognate sigma factor SigF [43]. Whether the interaction of Obg with UsfX affects the phosphorylation state of UsfX is unknown. Additional studies assessing the interaction of Obg and UsfX in vitro, and careful examination of phosphate exchange in vivo, may throw light on this part of Obg function. The Obg/CgtA proteins of *E. coli *and *V. harveyi *interact with SpoT, a stringent response regulator and a relative of RelA, which responds to starvation. The fact that Obg of *M. tuberculosis *fails to interact with RelA suggests that the stress response roles of Obg of *M. tuberculosis *differ from those of its homologues in other bacteria.

### Overexpression of Obg affects late log phase growth of *M. tuberculosis*

**Figure 5:**
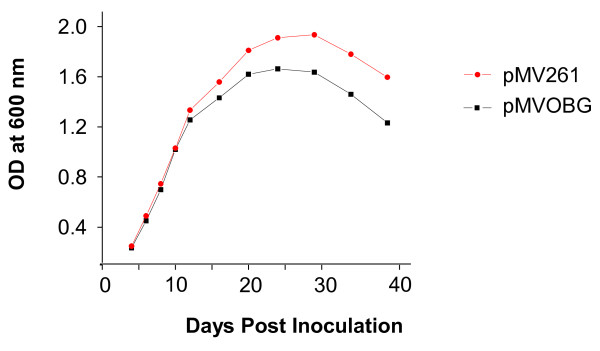
**Growth of *M. tuberculosis *strains at different time points**. *M. tuberculosis *was grown in 7H9-OADC-TW broth at 37°C. Growth was followed by measuring the OD at 600 nm using 1 ml aliquots. Closed circles: *M. tuberculosis *carrying the plasmid pMV261 (empty vector control); squares: *M. tuberculosis *carrying the plasmid pMVOBG (plasmid overexpressing Obg). The data shown are representative findings from three different. experiments.

Since expression of Obg in *M. tuberculosis *is growth regulated, we asked whether the presence of unusually high amounts of Obg might effect on the growth of this species. To do this, we followed the growth of *M. tuberculosis *strains bearing the Obg overexpression construct (pMVOBG), vs. strains containing the control plasmid (pMV261), over a period of time. Figure 5 shows that there is no significant difference in growth between the two strains during the early log phase, but that the growth of the Obg-expressing strain is decreased slightly in the late log phase, and that this relative decrease is continued even during the stationary phase (Figure 5). This indicates that overexpression of Obg suppresses cell division to some extent during the late log phase of *M. tuberculosis *growth. Similarly, increased expression of *E. coli *Obg, through an inducible promoter, suppresses log phase growth [11]. In contrast, overexpression of Obg has little effect on vegetative growth of *S. coelicolor*, but it significantly affects the development of aerial mycelia by this bacterium [9]. This and other examples have been used to support the proposal that an abundance of GTP-bound Obg is associated with vegetative bacterial growth (cell division), while a relative abundance of GDP-bound Obg promotes stationary development (viability in stationary growth, or differentiation leading to nonvegetative reproduction) [9].

## Conclusion

Our data reveal that *M. tuberculosis *Obg has characteristics that are common to its homologues in other bacteria, in addition to properties that are unique. Generation and characterization of mutant alleles of *M. tuberculosis *Obg should provide additional insights to the role of Obg in this important human pathogen, and toward identification of antimicrobials that reduce its ability to promote *M. tuberculosis *survival.

## Methods

### Bacteria and yeast strains and their growth conditions

*M. tuberculosis *H37Rv was grown either in Middlebrook 7H9 broth medium containing Tween (0.05%) and OADC (10%) (7H9-TW-OADC) broth, or in Middlebrook 7H10 agar medium containing Tween (0.05%) and OADC (10%) (7H10-TW-OADC). *M. tuberculosis *strains harboring plasmids were grown in the above media containing the antibiotic kanamycin (25 μg/ml) or hygromycin (50 μg/ml). *E. coli *strains containing plasmids were grown in LB broth or LB agar plates with the antibiotic(s) ampicillin (100 μg/ml), kanamycin (25 μg/ml) or both. Unless specified, all bacteria were grown at 37°C. The yeast strain AH109 was grown at 30°C in YPD broth or in agar supplemented with adenine hemisulphate (0.003%).

### DNA manipulation

Chromosomal DNA of *M. tuberculosis *H37Rv was isolated using cetyl trimethyl ammonium bromide (CTAB). Plasmid DNA from *E. coli *was isolated using Qiaprep kit (Qiagen Inc.). PCR reactions were performed as described by Ausubel et al [45], with genomic DNA of *M*. *tuberculosis *H37Rv used as the template for amplifying coding regions of its genes. Oligonucleotide primers (Table 2) were synthesized at the Center for DNA Technology at The University of Texas Health Science Center at San Antonio.

**Table 2:** List of primers used in this study.

Primer name	Primer sequence	Gene
TBOBG1	CCGCATATGAAGGGGAGCTCGGTGCCT CGG	*Obg*
TBOBG2	CGTCCGGATCCGGACTTCTCATCAGCCATCCCC	*Obg*
TBOBG5	CCGCAGGATCCGCACACTCCGCAGATGAAGGGGAGCTCGGTG	*Obg*
TBOBG6	ATGAAGGGATCCTCGGTGCCTCGGTTTGTCGATCGGGTC	*Obg*
TBRELAF	ACGCATATGGCCGAGGACCAGCAGCTCACGGCGCAAGCG	*RelA*
TBRELAR	ATGGGATCCTGCGTCTGCTCGGCGGAGAAAAGCGCG	*RelA*

Underlined nucleotides indicate the restriction sites created in the primers. CATATG, NdeI and GGATCC, BamHI.

To generate an Obg overexpression construct, we amplified the whole gene coding for Obg of *M. tuberculosis *by PCR with primers TBOBG1 and TBOBG2. These primers were designed to have an NdeI site at the 5'nd (TBOBG1) and a BamHI site at the 3'nd (TBOBG2). The DNA fragment obtained was cut with NdeI and BamHI and ligated to a similarly cut pET16b vector to create the plasmid pTBOBGE. In addition, we created several other plasmids to express Obg or other proteins in mycobacteria or yeast. Of these, plasmid pMVOBG was created to express Obg through a multicopy plasmid in mycobacteria. For making this plasmid, we first amplified the DNA fragment containing the coding region of Obg of *M. tuberculosis *by PCR, using the primers TBOBG5 and TBOBG6. The amplified DNA fragment was cut with BamHI and cloned into the BamHI site of pMV261 [46] downstream of the *hsp60 *promoter. Plasmid pGB2440c, for Obg expression in yeast, was created by cloning the NdeI-BamHI fragment containing *obg *from pOBGE into NdeI-BamHI-cut pGBKT7. Finally, plasmid pGA2853c, for RelA expression in yeast, was created by cloning the NdeI and BamHI cut DNA fragment containing the *relA *gene (*Rv2853*) amplified using primers TBRELAF and TBRELAR, into pGADT7. The cloned DNA fragments in all plasmids were verified by DNA sequencing for their appropriateness. All plasmids that we used in this study are described in Table 3.

**Table 3:** List of plasmids used in this study.

Plasmid	Description	Reference/source
pCR2.1	*ori*ColE1, *lacZα*, P*lac, aph*, Amp^R^	Invitrogen
pMV261	*ori*E, *oriM*, P*hsp60*, *aph*	Stover et al, 1991
pMVOBG	pMV261-*Rv2440c *full *orf*	This study
pET16b	*oriE, lacI*, P*T7*, Amp^R^	Novagen
pTBOBGE	pET16B-*Rv2440c full orf*	This study
pGADT7	*ori*ColE1, *ori*2 μ, *LEU*1, P_ADH1_::*GAL*4' activator domain::MCS Amp^R^	Clontech
pGBKT7	*ori*ColE1, *ori*2 μ, *TRP1*, P_ADH1_::*GAL*4' binding domain::MCS Km^R^	Clontech
pGADT7-T	SV40 large T-antigen_(84-708) _in pGADT7	Clontech
pGBKT7-53	Murine p53_(72-390) _in pGBKT7	Clontech
pGBKT7-Lam	Human lamin C_(66-230) _in pGBKT7	Clontech
pGA2853c	pGADT7-*Rv2853c *full *orf*	This study
pGB3286c	pGBKT7-*Rv3286c *full *orf*	Parida et al, 2005
pGA3287c	pGADT7-*Rv3287c *full *orf*	Parida et al, 2005
pGB2440c	pGBKT7-*Rv2440c *full *orf*	This study

### Overexpression of *M. tuberculosis *Obg in *E. coli *and production of antiserum

The *E. coli*-overexpressed Obg protein of *M. tuberculosis *was purified in its native condition. The plasmid construct pTBOBGE was transformed into *E. coli *strain BL21(DE3). A single transformant colony was selected and grown in 2 ml of LB broth overnight. One ml of this overnight culture was inoculated into 250 ml LB broth and grown to log phase (0.350 OD at 590 nm) at 37°C. IPTG (1 mM) was then added to the culture to induce overexpression of Obg, and the culture was grown for an additional 3 h. Afterwards, *E. coli *cells were harvested by centrifugation (5,000 g for 10 min at 4°C) and stored overnight at -80°C. The pellet was resuspended in 5 ml of lysis buffer (50 mM NaH_2_PO_4 _pH 8.0, 300 mM NaCl, 10 mM Imidazole) containing 1 mg/ml of lysozyme, incubated on ice for 30 min and the cells disrupted by sonication. The lysate was centrifuged at 12,000 g, and the supernatant was loaded on to a 2 ml Ni-NTA column (Qiagen). After washing the column with 50 ml of wash buffer (50 mM NaH_2_PO_4 _pH 8.0, 300 mM NaCl, 20 mM Imidazole), the column- bound Obg protein (His_10-_Obg) was eluted with 2 ml of elution buffer (50 mM NaH_2_PO_4 _pH 8.0, 300 mM NaCl, 250 mM Imidazole). The eluted fraction was dialyzed against 2 L of 20 mM Tris-HCl pH 8.0 containing 5% glycerol. About 100 μg of the resulting purified Obg was used to immunize a single rabbit to generate polyclonal antiserum, according to standard protocols. The rabbit received two booster doses of similar amounts of protein at two week intervals before collecting the serum two weeks after the last booster dose.

### GTP crosslinking

Crosslinking of the Obg protein with GTP was done by mixing Ni-NTA-purified *M*. *tuberculosis *His-tagged Obg (His_10_-Obg) (5 μg) with a 40 μl cross-linking mixture (20 μCi of [α^32^P]-dGTP, 1 mM ATP, 50 mM Tris HCl (pH 8.0), 100 mM NaCl, 5 mM MgCl_2 _and 1% Triton X-100). Eppendorf tubes containing the mixture were kept for 1 h at 4°C in a dark chamber, and then placed on ice over a Petri dish to expose them to UV light (256 nm) for different time periods. Crosslinking of Obg with GTP was assessed after separating the crosslinked complexes on SDS-PAGE, transferring the proteins from the gel onto nitrocellulose membranes, and exposure of the membranes to X-ray film to detect the presence of ^32^P in the protein bands.

### GTPase activity of Obg

To determine whether *M. tuberculosis *can hydrolyze GTP, we added [γ-^32^P]GTP to purified His_10-_Obg, following the method of Welsh et al [13]. The reactions were conducted in 100 μl volumes containing 50 mM Tris pH 8.5, 0.1 mM EDTA, 1.5 mM MgCl_2_, 200 mM KCl, 10% glycerol, 25. μCi of [γ-^32^P]GTP and 7 μg of His_10_-Obg. These reactions were incubated at 37°C for 3 h, and then terminated by the addition of 700. μ1 of ice cold 20.mM phosphoric acid (pH2. 0) containing 5% activated charcoal. The charcoal was sedimented by centrifugation, and 100 μl of the remaining supernatant used to measure the ^32^P_i _released. GTPase activity was expressed as ^32^P_i _released (cpm)/μg protein/hour.

### Autophosphorylation assay

To determine whether *M. tuberculosis *Obg is autophosphorylated in the presence of GTP, His_10_-Obg (5 μg) was incubated with 10. μCi of [γ-^32^P]GTP in a 25 μl reaction mixture containing 50 mM Tris-HCl pH 8.0, 0.1 mM EDTA, 1.5 mM MgCl_2_, 100 mM KCl and 10% glycerol at 37°C. The reactions were arrested at different time points by the addition of SDS-PAGE sample buffer. The samples from different time points were subjected to SDS-PAGE and transferred to nitrocellulose membranes, and autophosphorylation of the Obg protein was visualized by autoradiography.

### Soluble and membrane fractions

Soluble and membrane fractions of *M. tuberculosis *were prepared as described [47]. Briefly, *M. tuberculosis *cells were grown to 0.6-1.0 OD (at 600 nm) in 400 ml of 7H9-OADC-TW broth. The cells were then harvested by centrifugation at 5,000 g. The pellet was resuspended in 25 ml of 20 mM sodium phosphate-10 mM EDTA (pH 7.0) buffer, and spun again at 5,000 g to remove the medium completely. The pellet was then suspended in 4 ml of 20 mM sodium phosphate-10 mM EDTA buffer containing a protease inhibitor cocktail (Sigma), and divided into four 2 ml screw cap tubes with O-rings containing silica beads. The tubes were cooled on ice, and then the cells were disrupted using a bead beater for two 1-min cycles, with a 30 second interval between them. The tubes were chilled on ice for 5 min and then centrifuged at 12,000 g at 4°C for 15 min. The resulting supernatants were pooled, transferred to 4 ml centrifuge tubes and spun at 49,000 g for 4 h at 4°C. These supernatants (soluble fraction) were transferred to fresh tubes for analysis, while the pellet (membrane fraction) was washed once with 4 ml of 20 mM sodium phosphate-10 mM EDTA buffer and resuspended in 0.5 ml of the same buffer. Protein concentrations in both the soluble and membrane fractions, and in the unseparated lysates, were determined by the BCA method (Pierce) before subjecting them to electrophoresis.

### Preparation of ribosomal fractions

*M. tuberculosis *H37Rv cells were grown in 100 ml of 7H9-TW-OADC broth at 37°C. When the OD of the cultures reached to 0. 6 -1.0 (at 600 nm), the cells were harvested by centrifugation, resuspended in 2 ml of buffer A (10 mM Tris-HCl, pH 7.6, 10 mM magnesium acetate, 100 mM ammonium acetate_, _6 mM β-mercaptoethanol, and 2 mM PMSF), and disrupted by bead beating as described earlier. The lysate was then centrifuged at 12,000 g for 15 min. The clear supernatant was collected and its protein concentration determined. About 500 μg of this protein was loaded onto a 10-40% sucrose gradient (total volume 4 ml) made in buffer B (10 mM Tris-HCl, pH 7.6, 1 mM magnesium acetate, 100 mM ammonium acetate, 6 mM β-mercaptoethanol, and 2 mM PMSF). The gradient was centrifuged at 90,000 g for 20 h. The gradients were then aliquoted into 250 μl fractions, and the absorbance of each fraction measured (manually) at 260 nm. Magnesium acetate (10 μl of 1 M) was added to each fraction to increase the concentration of magnesium ions to 20 mM. The fractions were then mixed with equal amounts of 100% of ice-cold ethanol, and their proteins precipitated overnight at -80°C. The precipitates were collected by centrifugation at 12,000 g for 30 min. The pellets were resuspended in 100 μl of buffer A. Forty μl of the suspension from each fraction was mixed with 10 μl 4× loading buffer and boiled, after which 25 μl of each sample was loaded onto each well for SDS-PAGE. After electrophoresis, the proteins were transferred to nitrocellulose membranes, probed with anti-Obg antiserum, and the blots probed by ECL chemiluminescence method (Amersham). Association of Obg with ribosomal subunits was determined by comparing the immunoblot for each fraction with its absorbance at 260 nm.

### Yeast two-hybrid assay

Protein-protein interactions were performed using the Matchmaker Gal4 two-hybrid system 3 (Clontech, Palo Alto, CA) as described previously [42]. The yeast strain AH109, which has the reporter genes *ADE2 *(adenine), *HIS3 *(histidine), and *MEL1 *(α-galactosidase), was used as the host strain. Yeast plasmids (Table 2) were transformed into AH109 in appropriate combinations (Table 1) using standard protocols provided by Clontech. Expression of proteins by plasmids created for yeast two-hybrid analysis was assessed by the TNT Quick transcription and translation system (Promega), before transformation of the plasmids into yeast. Protein-protein interactions were determined by positive growth of yeast in synthetic drop out medium (SD) plates lacking adenine and histidine, and by the presence of blue color, which identifies α- galactosidase activity. To rule out false activation of the reporter gene, we transformed each of the constructs separately into yeast strain AH109, and assessed reporter gene activation. The strength of the interaction was verified by measuring the α-galactosidase released into the growth medium, again using protocols provided by Clontech.

### SDS-PAGE and immunoblot

SDS-PAGE and immunoblotting were performed following the methods of Ausubel et al [45]. Protein contents in extracts of *E. coli *or *M. tuberculosis*, obtained through sonication or bead-beating techniques, were determined by BCA (bicinchoninic acid) method (Pierce). Proteins were separated on 12% SDS-PAGE and transferred to nitrocellulose membranes. The blots were probed with rabbit anti-*M. tuberculosis *Obg antiserum (1:500 dilution) or rabbit anti-*M*. *tuberculosis *SigH antiserum (1:1000), developed against recombinant His_10-_Obg or His_10-_SigH proteins, respectively. Alkaline phosphatase-conjugated anti-rabbit IgG (Zymed, 1:1000 dilution) or peroxidase-conjugated anti-rabbit IgG (Sigma, 1:10,000 dilution) were used as secondary antibodies. The blots were developed either with 5-bromo-4-chloro-3-indolyl phosphate (BCIP)/nitroblue tetrazolium (NBT) substrate (Sigma, for alkaline phosphatase), or with an ECL kit (Amersham, for peroxidase).

## Authors' contributions

**SJS **performed the construction of plasmids and isolation of ribosomal fractions. **SS **carried out the overexpression of Obg and its biochemical analysis. **VLS **read the manuscript critically, participated in interpretation of the data, and worked with the other authors to prepare the final version of the paper. **SD **conceived the study, participated in its design and interpretation of results and wrote the manuscript. All authors read and approved the manuscript.

## Supplementary Material

Additional file 1 **Amino acid alignment of Obg proteins from different bacterial species**. MTOBG, *Mycobacterium tuberculosis *Obg; SCOBG, *Streptomyces coelicolor *Obg; BSOBG,*Bacillus subtilis *Obg; ECOBG, *Escherichia coli *ObgE; CCOBG, *Caulobacter crescentus *Obg (CgtA). Asterisks (*) indicate high amino acid identity, colons (:) indicate medium amino acid identity, and dots (.) indicate low amino acid identity. GTP-binding motifs G1, G2, G3, G4, switch I and switch II are marked. Click here for file

Additional file 2 **SDS-PAGE analysis of total proteins associated with different ribosomal fractions**. Ribosomal fractions (1-15) from wild-type *M. tuberculosis *extracts were separated on a 10%-40% sucrose gradient. *M. tuberculosis *was grown in 7H9-OADC-TW broth at 37°C, and extracts for ribosomal isolation prepared using a bead beater. Five hundred μg of protein was separated in 10-40% sucrose gradient by centrifugation. The sucrose gradient was then aliquoted into 250 μl fractions and their ODs measured at 260 nm. The proteins in the fractions were precipitated with ethanol and separated on SDS-PAGE, stained with Coomassie blue and destained with 10% acetone. The gel picture shown here is modified from its original to eliminate and correct mis-loaded and incorrectly loaded lanes. Click here for file
