# Supplementary figures and images for: Biochemical and physiological characterization of the GTP-binding protein Obg of Mycobacterium tuberculosis
Source: BMC Microbiol. 2011 Feb 25;11:43. doi: 10.1186/1471-2180-11-43 (PMC3056739; doi:10.1186/1471-2180-11-43)

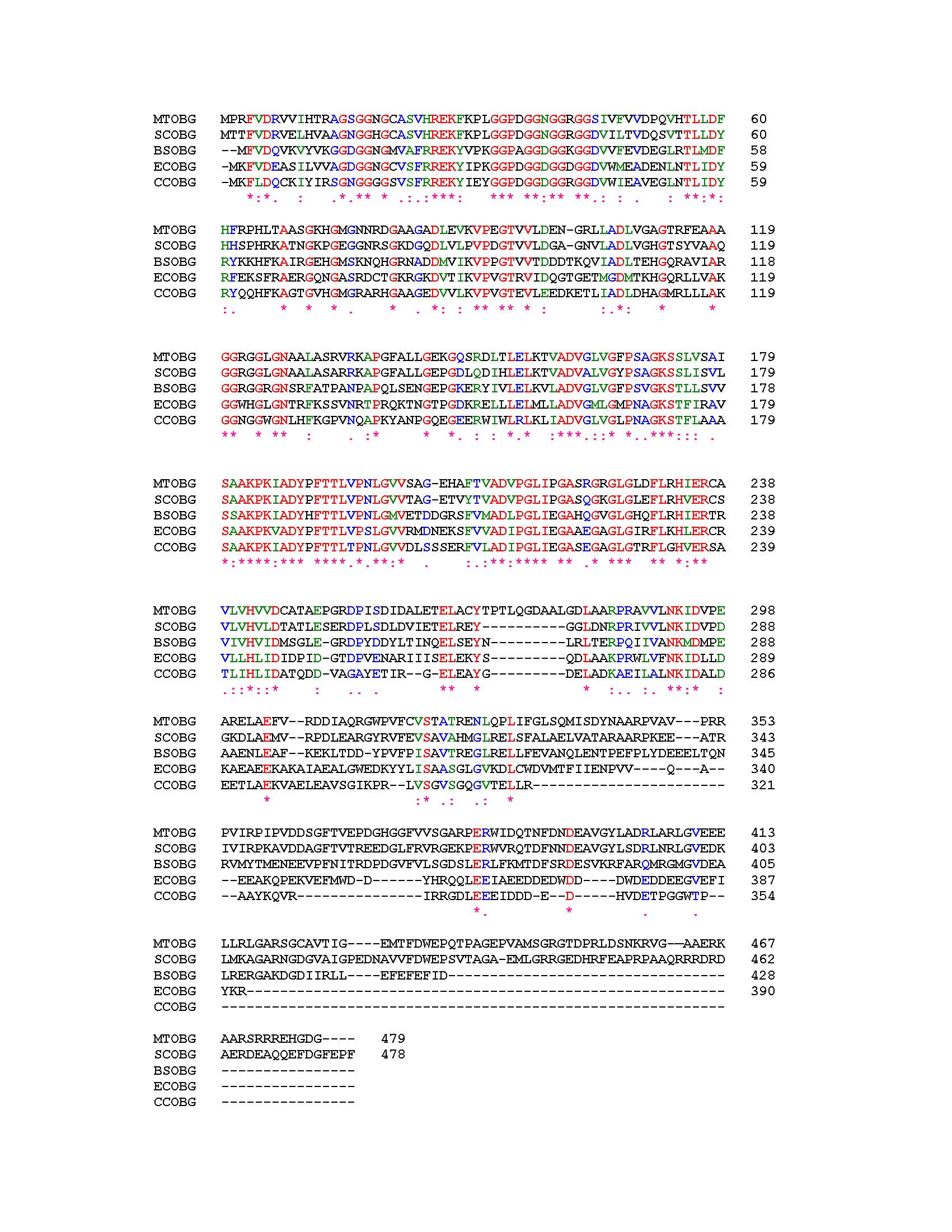


**G1**

**G2/Switch I**

**Switch II**

**G4**

**G3**

Supplement: Additional file 1 — Amino acid alignment of Obg proteins from different bacterial species. MTOBG, Mycobacterium tuberculosis Obg; SCOBG, Streptomyces coelicolor Obg; BSOBG,Bacillus subtilis Obg; ECOBG, Escherichia coli ObgE; CCOBG, Caulobacter crescentus Obg (CgtA). Asterisks (*) indicate high amino acid identity, colons (:) indicate medium amino acid identity, and dots (.) indicate low amino acid identity. GTP-binding motifs G1, G2, G3, G4, switch I and switch II are marked. [file 1471-2180-11-43-S1.DOC]

## Slide 1
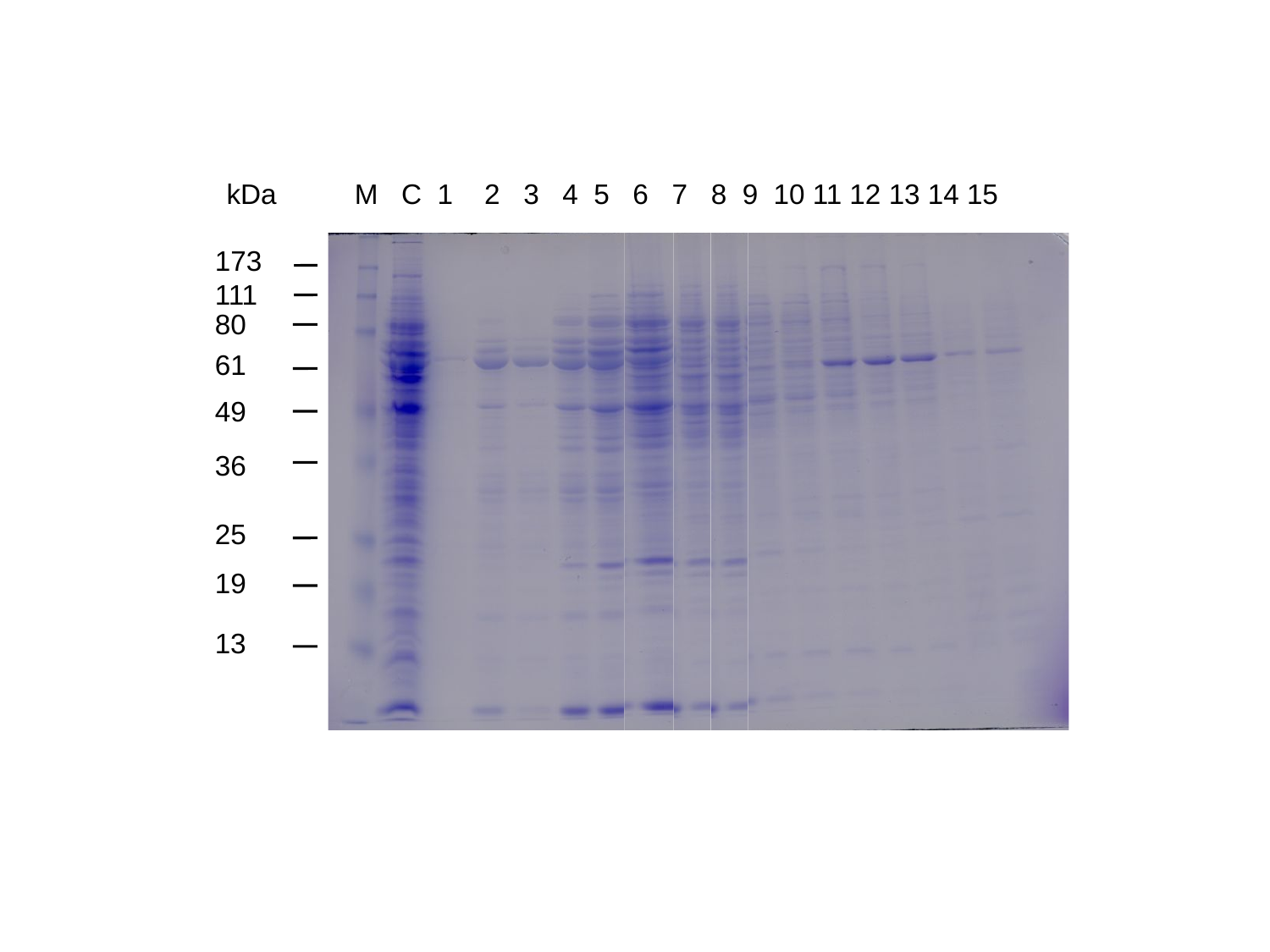

kDa M C 1 2 3 4 5 6 7 8 9 10 11 12 13 14 15
173
111
80
61
49
36
25
19
13

Supplement: Additional file 2 — SDS-PAGE analysis of total proteins associated with different ribosomal fractions. Ribosomal fractions (1-15) from wild-type M. tuberculosis extracts were separated on a 10%-40% sucrose gradient. M. tuberculosis was grown in 7H9-OADC-TW broth at 37°C, and extracts for ribosomal isolation prepared using a bead beater. Five hundred μg of protein was separated in 10-40% sucrose gradient by centrifugation. The sucrose gradient was then aliquoted into 250 μl fractions and their ODs measured at 260 nm. The proteins in the fractions were precipitated with ethanol and separated on SDS-PAGE, stained with Coomassie blue and destained with 10% acetone. The gel picture shown here is modified from its original to eliminate and correct mis-loaded and incorrectly loaded lanes. [file 1471-2180-11-43-S2.PPT]
